# Dynamic Ileus Following Operations Involving the Abdominal Cavity, with Remarks on Adynamic Ileus1Read before the American Association of Obstetricians and Gynecologists at Niagara Falls, August 19, 1897, and published in the *Annals of Gynecology and Pediatry*.

**Published:** 1897-10

**Authors:** F. Blume

**Affiliations:** Allegheny, Pa., Gynecologist to the Allegheny General Hospital and Pittsburg Free Dispensary; obstetrician to the Roselia Maternity Hospital; consulting gynecologist to the Mercy Hospital; Fellow American Association of Obstetricians and Gynecologists


### BUFFALO MEDICAL JOURNAL.

VOL. XXXVII. OCTOBER, 1897. No. 3.

### Original Communications.

### DYNAMIC ILEUS FOLLOWING OPERATIONS INVOLVING THE ABDOMINAL CAVITY, WITH REMARKS ON ADYNAMIC ILEUS.[1]

By F. BLUME, M. D., Allegheny, Pa.,

Gynecologist to the Allegheny General Hospital and Pittsburg Free Dispensary; obstetrician to the Roselia Maternity Hospital; consulting gynecologist to the Mercy Hospital; Fellow American Association of Obstetricians and Gynecologists.

INTESTINAL obstruction following operations involving the abdominal cavity has within the past few years been frequently the subject of discussion in societies and journals. Quite a number of cases have been reported and from the evidence which they present we are led to conclude that this grave complication is by no means of such rare occurrence as hitherto believed, and that it demands our earnest consideration to correct old views and to establish more precise indications, which may guide us in diagnosis and treatment.

A decided progress in this direction was made when Olshausen,[2] in 1887, in an article read before the Berlin Obstetrical and Gynecological Society, called attention to a form of intestinal obstruction which had not yet been recognised, a paralysis of the intestine not dependent upon septic peritonitis. The symptoms in these cases are: temperature normal or but slightly elevated, in some instances even subnormal; pulse-rate increased, some vomiting. On the second or third day after operation, or even later, the symptoms of collapse manifest themselves. The pulse is rapid and feeble, the abdomen becomes more and more distended, retching and vomiting more frequent. Neither gas nor feces are expelled

1. Read before the American Association of Obstetricians and Gynecologists at Niagara Falls, August 19, 1897, and published in the *Annals of Gynecology and Pediatry*.

by the rectum. The general condition of the patient during the first few days appears to be good. Nothing is observable by which the development of so serious a complication can even be suspected until the abdominal symptoms become more pronounced. Death ensues between the fourth and tenth day after the operation and results from intoxication of the decomposed intestinal contents. Post mortem examination reveals the absence of peritonitis. Olshausen sees in prolonged eventration of the bowels an important etiological factor of this condition.

Notwithstanding the fact that competent observers have confirmed the investigations of Olshausen, there is still difference of opinion in regard to the causes of this variety of adynamic ileus. The opponents of this theory are not satisfied with the ordinary post mortem examination ; they insist on bacteriological examinations to exclude sepsis. It is not my intention to enter upon a discussion of this theory. I will but briefly refer to an interesting article of Otto Engstrem,[3] published this year. This author gives his experience with four cases of intestinal paralysis following abdominal operations. Everyone of the patients died, although in two of them a secondary operation was performed. Post mortem examinations showed the absence of peritonitis. In the last case a careful bacteriological examination was made with negative result. Reviewing the investigations of other observers, he arrives at the conclusion that paralysis of the intestines can and does occur after operations involving the abdominal cavity without infection at the time of the operation. Eventration and prolonged manipulations of the bowels are the most potent etiological factors, causing irritations of the nerves of the mesentery and the gutwall and leading to changes in the circulation. From his own experience he can confirm the statement of other observers that a paresis of the intestinal wall can be produced by strong saline purgatives, and it appears to be by no means impossible that, as a consequence of their use, a weakened condition of the gutwalls is created before the operation. He looks upon the increased susceptibility of the nervous system, often so marked in patients before operation, as a predisposing etiological factor.

There is hardly a surgeon who cannot recall a case in which the symptoms described above presented themselves, where the prognosis seemed to be favorable during the first few days and which ended fatally from intestinal obstruction before a week had passed by. The diagnosis remained uncertain ; even the autopsy did not give a satisfactory explanation. Such a case came under my observation in April, 1896. The patient, from whom I had removed a right pyosalpinx, was very restless after operation and could scarcely be managed. General condition good on the second and third day. In the middle of the fourth day the symptoms of intestinal obstruction developed; temperature, 99°; pulse, 130. Peristalsis was perceptible from the second to the fourth day. Collapse in the morning of the fifth day; temperature, 97 3-5°; pulse, 142. Death towards the end of the fifth day. Autopsy: the intestines enormously distended, their peritoneal covering slightly injected, but otherwise normal in appearance. No adhesions, no evidence of peritonitis. This post mortem examination made a deep impression upon me.

I have presented to you this brief sketch of one type of adynamic ileus to bring out certain points and conditions which more or less are associated with every form of intestinal obstruction and to which reference will have to be made repeatedly later on. It may serve, therefore, as an introduction to the study of dynamic ileus, to which I now invite your attention.

The first case of dynamic ileus following operation which I have been able to find in the literature at my disposal, occurred in 1887 in Olshausen's clinic and was published in 1888 by P. Reichel,[4] one of his assistants. It was a case of vaginal hysterectomy for cancer of the cervix uteri. The patient did well the first days. No rise of temperature. Nausea, but no vomiting. The pulse-rate remained above normal and increased to 140 on the third day. Abdomen distended, marked peristalsis, visible through the thin abdominal walls. No gas or feces expelled by the rectum. Enemata and stomach-washing gave no relief. The abdomen was opened on the seventh day. The small intestines were greatly distended, their serous coat strongly injected, but smooth and glistening. The lower part of the ileum, about five inches from the ileo-cecal valve, and a portion of the mesentery were adherent to the vaginal wound. On separating the adhesions three to three and one-half inches of this part of the bowel were found to be firmly contracted, resembling a solid cord. All efforts to move the intestinal contents along by compression and thus dilate the contracted portion failed. The patient died on the table.

Numerous cases of intestinal obstruction after operation have been observed in which the ante- or post mortem examination showed that a few inches of the gut were "collapsed" or "contracted." Either a constriction by peritoneal bands, or an acute flexure, or some other mechanical obstruction has been given as a causative factor; they, therefore, do not belong to the form of obstruction now under consideration.

I have found but two more cases recorded which illustrate the dynamic variety of ileus following operation—the cases of my friend Dr. X. O. Werder. They are well known to you as they were made a part of the exceedingly interesting paper which Dr. J. W. Long[5] presented to this association at the Richmond meeting last year.

I wish to submit to your consideration today a brief report of a case of dynamic ileus which I saw early last fall.

Mrs. M., aged 36 years, mother of two children, very nervous and emaciated. Large unilocular intraligamentous cyst of right ovary, adherent to bowel and omentum.

Ovariotomy, September 29, 1896. Patient was very weak after the operation, but reacted well after the use of stimulants. Nausea and vomiting during the first twenty-four hours. Slight elevation of temperature; pulse-rate remained above normal. On the second day she was given and retained small doses of hot water and broth. Gas was expelled by the rectum. Her general condition improved and at the end of the second day with normal pulse and temperature, she seemed to be quite comfortable and the case looked very favorable. In the following night, early in the morning, she again commenced to vomit and for the first time complained of colicky pain in her abdomen, which was slightly distended. Increased peristalsis was audible; gurgling sounds could be heard at the bedside. Her condition grew gradually worse towards the end of the third day. The temperature varied between 99° and 101°, the pulse-rate between 100 and 120. All attempts to obtain a movement of the bowels by cathartics and intestinal injections resulted in failure. The distention of the abdomen increased. The patient became very nervous and restless and the symptoms of collapse began to manifest themselves. Fecal vomiting occurred about seventy-six hours after operation. Half an hour later I found her profoundly collapsed with a pulse of 150, hardly perceptible, no peristaltic action of the bowels ascertainable. The diagnosis of intestinal obstruction dependent upon some mechanical cause seemed to be certain.

The patient having been etherised I reopened the incision. The small intestines were very much distended, their peritoneal covering slightly injected, smooth and glistening. Six inches above the ileocecal valve and for a distance of about ten inches the ileum was firmly contracted, its lumen completely obliterated. The contracted portion was strongly wrinkled as if a purse-string suture had been applied and tightly drawn. At its proximal end there was a slight abrasion. Immediately above the contracted portion the bowel was enormously distended and sacculated, showing the effect of the violent peristaltic action. The bowel below it was soft, empty and collapsed. No adhesions, no fluid in the abdominal cavity, no evidence of peritonitis.

An attempt to force gas into the contracted portion by compressing the bowel above it was not successful. I opened the ileum and a large amount of gas came away. Having stitched the bowel, I again turned my attention to the contracted portion, which was found to be unchanged. By this time the patient was almost pulseless, and I had to close the abdomen quickly. She recovered from the anesthetic and appeared to be improving for several hours, when, early in the morning, coma supervened. She died in the middle of the fourth day after the first operation. No autopsy.

I regret to say that I have nothing to offer which could satisfactorily explain dynamic ileus. When I saw the firmly contracted portion of the ileum it at once reminded me of the experiment conducted by Dr. Robert T. Morris[6] at the Toronto meeting of this association, while demonstrating the mechanism of ileo-intussusception in a rabbit. A sudden spasm of the circular muscle of the bowel was produced by touching the ileum with a bit of carbonate of sodium. The caliber of the bowel was decidedly reduced and intussusception occurred.

From the evidence which this experiment and the cases under discussion present it seems to be at least not improbable that certain toxic substances, the result of decomposition of the intestinal contents play the most important part in the etiology of dynamic ileus. I look upon them as the exciting causes, which under certain circumstances may produce tonic contractions, a tetanic condition of a segment of the intestine, which not only continues under anesthesia but, at least for hours, even after death. This contraction, as the reports show, is found in those parts of the bowel which are nearest to the field of operation and exposed to manipulations and injuries. Here a point of least resistance is created in the intestinal tube by the irritations of the nerves supplying this part, by the disturbance of their mechanism and by the changes which take place in the gutwall.

I do not attempt an explanation of the very extensive tonic contractions found in Dr. Werder's first case. As in the other cases the lower portion of the ileum was involved, but besides this the whole large intestine was in a state of firm contraction, a condition which I think it is impossible to satisfactorily explain at the present time. Further investigations into the pathology of dynamic ileus are needed to solve this and allied questions.

I beg to direct attention to the fact that in none of the four cases on record has a correct diagnosis been made. In my own case I arrived at the opinion that it was a mechanical obstruction I had to deal with. The symptomatology of this case is indicative of the presence of an obstruction, probably in the lower portion of the small intestine, but nothing more. Whether this obstruction was due to a tonic contraction, or to a flexure, or constriction, or any other form of mechanical obstruction, could not be determined.

As stated in the report, the patient was doing well at the end of the second day and during the following night until in the morning, when she complained of pain in the abdomen, was nauseated and began to vomit. When I saw her in the forenoon the abdomen was slightly tympanitic, peristalsis increased, pulse accelerated. The pain complained of was not limited to a certain point, but extended over the whole abdomen and was thought to be dependent upon the active peristaltic movements of the intestines. These conditions are so familiar to us; we meet with them so often in favorable cases at this period after operation that we do not regard them as unusual. We know them to improve quickly after a movement of the bowels is obtained.

I saw the patient again in the afternoon. Her condition had grown worse. The inability to get the bowels to act, the increased peristalsis and the manifestations of threatening collapse suggested the probability of intestinal obstruction. The advisability of reopening the abdomen was carefully considered, but the diagnosis was by no means clear at this time to justify interference.

It is probably more interesting to the surgeon than important to the welfare of the patient, to attempt to differentiate in cases of post-operative intestinal obstruction between the various forms of ileus. Dynamic and mechanical ileus, to which it clinically belongs, as well as the adynamic variety, which I have described in my introductory remarks, and the ileus of sepsis, all lead to the same end—death—unless relieved by timely surgical interference. For practical purposes such differential diagnosis is hardly needed. We must distinguish, however, between these forms of ileus and the very obstinate constipation, often so difficult to overcome after operations. This differential diagnosis is extremely difficult early in the course of the disease, in those instances where the symptoms are less distinct. Even fecal vomiting does not remove the doubt, for it sometimes occurs in favorable cases, *i. e.*, in cases which get well without surgical interference. It was not present in Olshausen's cases of adynamic ileus and occurred late in my case of dynamic ileus.

There can be no doubt that these cases of obstinate constipation represent a mild form of that type of adynamic ileus described by Olshausen. In some instances, however, we must regard them as the result of an infection, of a slight localised peritonitis, which quickly subsided. As in cases of septic ileus, peristalsis is absent or greatly diminished, even hardly perceptible. This most important symptom which distinguishes obstinate constipation and the various forms of adynamic ileus from dynamic and mechanical ileus is apparently often overlooked or its value underrated.

During the progress of the disease the abdominal symptoms become more pronounced, the pulse more accelerated. The patient grows nervous and restless, she seems to realise that her life is in danger. Clearly, this condition can no longer be confounded with that of obstinate constipation. The only question to decide at this advanced stage is the differential diagnosis between septic peritonitis and ileus. The general condition of the patient during the first few days, the absence or slight elevation of temperature and the character and rate of the pulse at this time permit us to exclude sepsis as the probable lesion. The continued increased peristaltic action confirms this view and makes it clear that an occlusion of the bowel exists.

We have been, and still are, under the influence of that doctrine which sees in every case of intestinal obstruction subsequent to operation in the first place a case of septic peritonitis, whether there be a rise of temperature or not. It is the influence of this doctrine which has stilled the hands of many a surgeon until the patient was beyond the reach of human aid. So deeply rooted is this doctrine that even in those cases which die on the fourth day or later, and in which at the autopsy no visible evidence of peritonitis can be found, sepsis is diagnosticated, or at least suspected, and the operator criticises himself and his assistants for having infected the patient. It is well known that a patient may die of acute fulminating peritonitis in from twenty to thirty-six hours after operation without any symptoms of peritonitis in the abdominal cavity, but why these symptoms should not develop after sufficient time for their development has elapsed before death ensues is certainly difficult to comprehend. The true cause of death in these cases which terminate fatally at or after the fourth day is by no means clear. Is it not fair to assume that these patients do not die from peritonitis alone, if it be admissible to thus term this condition, but that death must be attributed far more to the toxic substances which result from decomposition of the intestinal contents?

Whenever a doubt remains as to the true nature of the disease, the patient should have the benefit of the doubt. The abdomen should be reopened, or in cases following vaginal hysterectomy an exploratory incision should be made. The gravity of the condition and the slight danger attending these methods of investigation render them justifiable.

The importance of an early diagnosis should never be lost sight of. Collapse may set in unexpectedly, making surgical interference hopeless. The early profound collapse in my case must be attributed to the very poor health of the patient, whose vitality was far below par when I first saw her. With the appearance of collapse peristalsis ceased, the intestines being over-distended and paralysed. There are no points of distinction between the various forms of ileus—including the paresis of general peritonitis—at this stage of the disease.

I have omitted to differentiate between dynamic ileus and the various types of mechanical ileus. Dynamic ileus belongs clinically to the mechanical variety, from which it cannot be distinguished if the obstruction occurs in the same portion of the intestine. A discussion of the accessory symptoms, which are observable in cases of mechanical ileus, and which differ according to the location and character of the obstruction, is deemed to be beyond the scope of this article.

Dynamic ileus following operations must be regarded as a surgical disease, and surgical treatment, in order to be successful, must be instituted before the vital forces of the patient are exhausted. This statement may at first sight seem to be too radical. The lesson, however, which the cases on record teach us, in my opinion, warrant this assertion, notwithstanding the fact that all the patients died, two with and two without operative interference.

The ordinary means—cathartics, enemata and stomach-washing, —have been faithfully tried and have utterly failed to give relief. The nature of the obstruction has led to the suggestion of antispasmodics. I do not think they can influence a contraction which persists under anesthesia, nor do I understand how we are to find the indication for their use, as post-operative dynamic ileus cannot be diagnosticated.

It is of the greatest importance not to lose time with a method of treatment which, though giving temporary relief, is not curative. I refer to stomach-washing, and I fully agree with those who see in such temporary improvement a danger to the patient, as it is apt to deceive the surgeon. From my own experience, I must warn against lavage in the later course of the disease, as it decidedly hastens collapse.

The two patients treated surgically died; they were operated on too late. As we know nothing about the condition of the contracted portion of the bowel after operation, it is questionable whether a simple incision of the intestine will save the patient. As an emergency operation, a portion of the distended bowel should be sutured to the abdominal wound, incised and the intestinal contents removed. If necessary, a secondary operation may be done later.

2. Zeitschrift für Geburtshülfe und Gynækologie, Vol. XIV., p. 619.

3. Zeitschrift für Geburtshülfe und Gynækologie, Vol. XXXVI., p, 399.

4. Zeitschrift für Geburtshülfe und Gynækologie. Vol. XV., p. 37.

5. Transactions of the American Association of Obstetricians and Gynecologists, Vol. IX., p. 214.

6. Transactions of the American Association of Obstetricians and Gynecologists, Vol. VII., page 198.

118 Liberty Street.

